# Successful Debulking of Tricuspid Valve Vegetation Using Suction Filtration and Veno-Venous Bypass

**DOI:** 10.7759/cureus.22741

**Published:** 2022-03-01

**Authors:** Arshan Khan, Moiz Ehtesham, Haris Asif, Maria Riasat, Kootaybah Alsheikhly

**Affiliations:** 1 Internal Medicine, Ascension St. John Hospital, Detroit, USA; 2 Internal Medicine, Albany Medical Center, Albany, USA; 3 Internal Medicine, Woodhull Medical Center, New York, USA; 4 Internal Medicine, Icahn School Of Medicine Mount Sinai Beth Israel, New York, USA; 5 Cardiology, Ascension St. John Hospital, Detroit, USA

**Keywords:** veno-venous bypass and suction filtration for infective endocarditis, veno-venous bypass and suction filtration, septic pulmonary emboli, right sided vs left sided infective endocarditis, indication for surgery in right sided infective endocarditis, right sided infective endocarditis, angiovac system

## Abstract

Tricuspid valve endocarditis with recurrent septic pulmonary emboli is an indication for surgery. In this report, we present a case of right-sided infective endocarditis (RSIE) in a female patient with a history of intravenous drug use (IVDU). The patient was admitted with multiple chief complaints of fatigue, chills, fever, cough, chest pain, and shortness of breath. She was found to have a large 1.8 cm (W) x 2.4 cm (L) mobile tricuspid valve vegetation on transthoracic echocardiogram (TTE). Despite being on appropriate antibiotics, the patient failed to improve clinically. Cardiothoracic surgery (CTS) evaluated the patient for surgical management of infective endocarditis (IE) given the size of vegetation, persistent bacteremia, and clinical deterioration. However, the risk/benefit ratio for open-heart surgery was high, given the history of active IVDU and hemodynamic instability. The patient underwent percutaneous extraction of the vegetation using suction filtration and veno-venous bypass and her condition significantly improved clinically afterward. We discuss the importance of suction filtration and veno-venous bypass in managing tricuspid valve endocarditis as an alternative in patients who are not ideal candidates for surgery and the need for more evidence regarding its effectiveness compared to surgery.

## Introduction

Right-sided infective endocarditis (RSIE) is frequently associated with intravenous drug use (IVDU) [1]. Compared with the extensive data on left‐sided infective endocarditis (IE), there is much less published information on the management of RSIE [2]. RSIE usually presents as fever with respiratory symptoms, and diagnosis requires a high index of suspicion [3]. Diagnosis can be confirmed by transthoracic echocardiography (TTE), except in patients with implanted cardioverter defibrillators, where a transesophageal echocardiogram (TEE) may be necessary [4].

The first-line treatment for tricuspid valve endocarditis is medical management [5]. In patients with RSIE, surgery is indicated by large-size vegetations (defined as >2 cm for the right side), if blood cultures remain positive despite being on appropriate antibiotics, or in cases of recurrent septic pulmonary embolism [6]. There are no clear-cut guidelines for surgery in patients with RSIE and active IVDU, as the risk of reinfection is higher with the prosthetic valve if the patient continues to use intravenous drugs and is non-compliant with medications [7]. We discuss the case of a patient with native tricuspid valve endocarditis with multiple septic pulmonary emboli, who underwent percutaneous extraction of the vegetation using suction filtration and veno-venous bypass and clinically improved afterward.

## Case presentation

**Figure 1:**
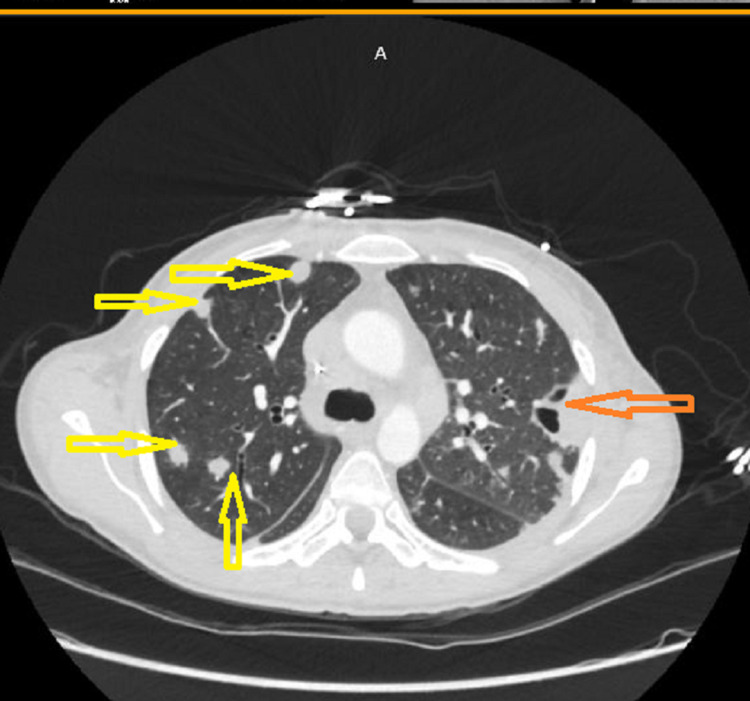
CT chest showing multiple septic pulmonary emboli (yellow arrows) and cavitary nodule (orange arrow) CT: computed tomography

A 36-year-old female with a past medical history of daily IV heroin use and mood disorder presented with chief complaints of shortness of breath, severe chest pain, cough productive of brown and black sputum, daily fevers, and chills for two weeks. On presentation, the patient's vital signs were as follows - blood pressure: 112/59 mmHg; heart rate: 129 beats/minute; and respiratory rate: 27 breaths/minute. On physical examination, the patient had dry mucous membrane, sunken eyes, decreased capillary refill and skin turgor, and a holosystolic murmur of tricuspid regurgitation; the exam was otherwise unremarkable. On admission, the laboratory results were as follows: white blood cell (WBC) count of 15,000 cells/mcL, a hemoglobin level of 8 g/dL, platelet count of 277,000 cells/mcL, and creatinine level of 0.4 mg/dl. A chest X-ray was performed and showed possible septic pulmonary emboli. Later on, a CT angiogram (CTA) confirmed septic pulmonary emboli (Figure 1). An ECG was remarkable for sinus tachycardia with a heart rate of 124 beats/minute and a short PR interval of 92 ms.

There was a concern for IE, and the patient was empirically started on vancomycin dosing per pharmacy protocol, piperacillin-tazobactam 4.5 gram IV every eight hours, and clindamycin 600 mg every six hours. TTE showed a large 1.8 cm (W) x 2.4 cm (L) mobile vegetation on the atrial aspect of the anterior leaflet of the tricuspid valve and moderate tricuspid regurgitation. Blood cultures revealed the growth of methicillin-resistant *Staphylococcus aureus* (MRSA). The patient also had right-knee swelling and tenderness. She underwent aspiration of the right knee. During the arthrocentesis, roughly 30 ml of grossly bloody and cloudy fluid with purulent-like debris was observed and sent for analysis. Synovial fluid cultures came back positive for MRSA. The patient's repeat blood cultures remained positive despite being on appropriate antibiotics for five days. As per cardiothoracic surgery (CTS) recommendations, the risk/benefit ratio for open-heart surgery was high, given the history of active IVDU and hemodynamic instability.

**Figure 2:**
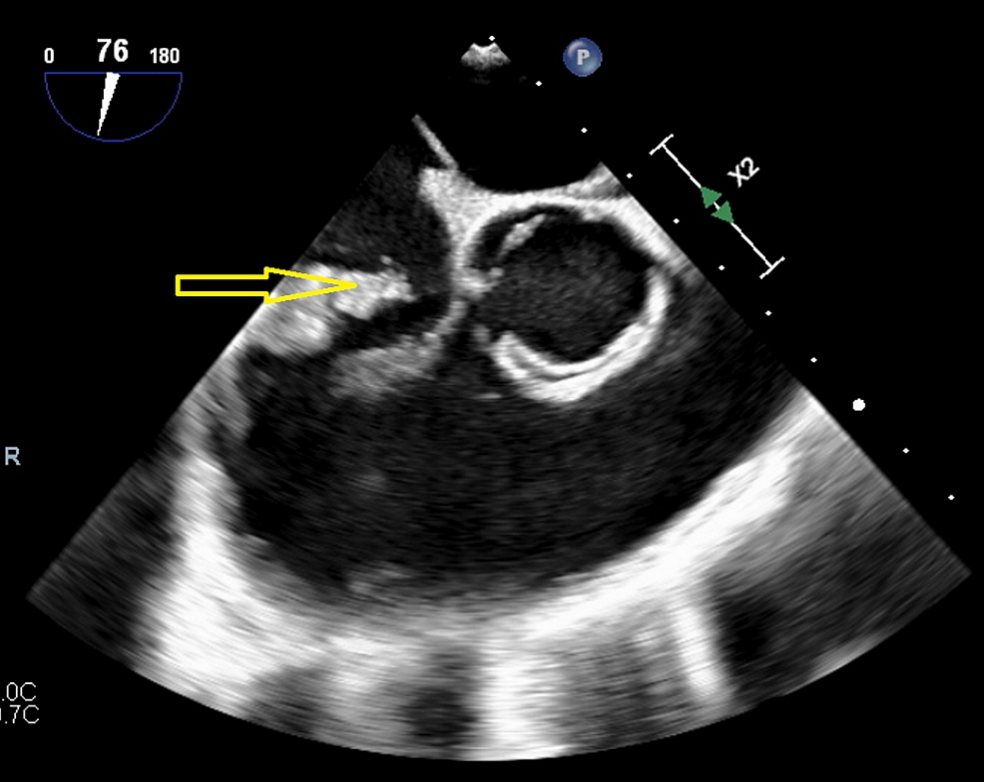
Short-axis view of TEE showing large vegetation attached to the anterior leaflet of the tricuspid valve (arrow) TEE: transesophageal echocardiogram

**Figure 3:**
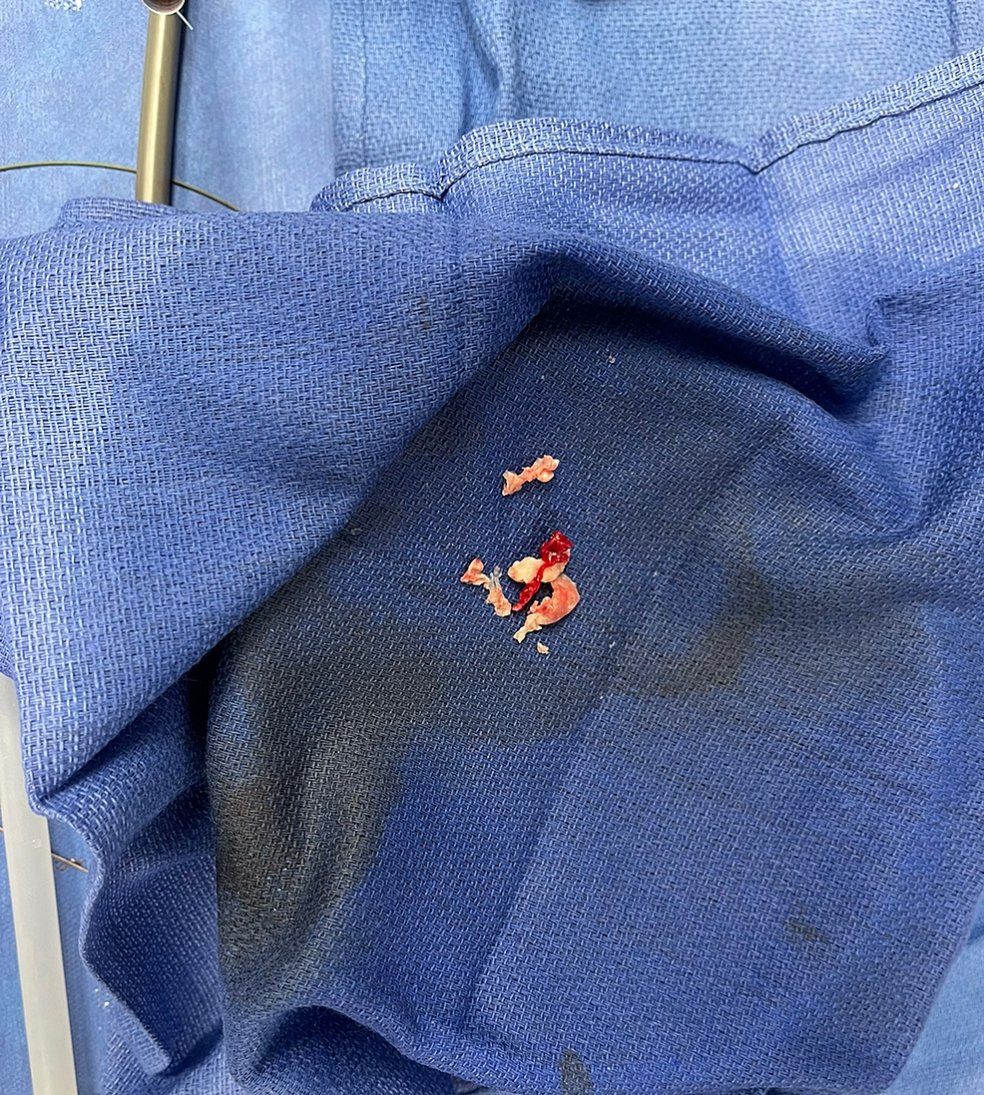
Tricuspid valve vegetation and debris post AngioVac suction

Subsequently, the patient underwent percutaneous extraction of the tricuspid valve vegetation using suction filtration and veno-venous bypass. The right internal jugular and right common femoral vein were used as an access, and the procedure was performed under TEE and fluoroscopy guidance. During the procedure, TEE showed moderate to severe tricuspid valve regurgitation and a 2.4 x 1.2 cm vegetation attached to the atrial side of the anterior tricuspid valve leaflet (Figure 2). The specimen was sent to the pathology lab (Figure 3). The patient became hemodynamically stable the day after the procedure, and she was not febrile anymore. She was transferred to the medical unit. Repeat blood cultures yielded no growth. The patient was counseled extensively about quitting substance abuse and the importance of medication compliance. No post-procedure echocardiogram was performed during the admission. Cardiology and infectious disease (ID) consultants deemed the patient stable for discharge. She was discharged to a rehab facility with six weeks of IV vancomycin and instructions for cardiology and ID follow-up. Unfortunately, the patient was lost to follow-up.

## Discussion

RSIE accounts for 5-10% of all cases of IE. It is predominantly encountered in the IVDU population, where HIV and hepatitis C virus infections often coexist [3]. Of note, 90% of RSIE involves the tricuspid valve [8]. RSIE differs from left-sided IE in clinical presentations, diagnostic findings, and prognoses. Pulmonary embolism and its related complications are common in RSIE, as shown in our case. In contrast, systemic emboli are common in left-sided IE [4]. The modified Duke's criteria are used to diagnose IE [9]. Positive blood culture and echocardiogram findings of IE are the major criteria for the diagnosis of IE. However, blood cultures can be negative in up to 30% of confirmed IE cases, placing greater emphasis on the role of cardiac imaging [10].

Most patients with tricuspid valve IE can be successfully treated with antibiotics; however, 5-16% of cases eventually require surgical intervention [8]. In patients with RSIE, surgery is indicated by large-size vegetations (defined as >2 cm for the right side), if blood cultures remain positive despite being on appropriate antibiotics, or recurrent septic pulmonary embolism [5-6,10]. The vegetation size helps to decide if the patient needs surgery or not. It is also an independent predictor of mortality and recurrence. Vegetation size >2 cm is an independent predictor of all-cause late mortality, and vegetation size >2.5 cm predicts late IE recurrence [11-12].

Surgical approaches for tricuspid valve surgery include replacement or repair. In general, valve repair is preferred over replacement; the approach depends on the extent of the damage and the available local expertise [5]. Surgery for patients with RSIE is a matter of controversy because of relapse due to non-compliance with medical therapy and recurrence due to ongoing IVDU [7]. In cases where a patient is not an ideal candidate for surgery (such as those who are critically ill with a high risk of perioperative mortality or relapse in the setting of active IVDU), there is a relatively new option for the management of RSIE where we can perform percutaneous extraction of the vegetation using suction filtration and veno-venous bypass [13-14].

The suction filtration and veno-venous bypass system require two access points: the venous return cannula and the reinfusion cannula. It may include any combination of femoral and or internal jugular vein. The venous return cannula is placed in an internal jugular; its size is typically 22 Fr and is placed under fluoroscopic guidance. The suction system is then inserted through the internal jugular vein cannula. Then, a suction force is generated with a bypass pump's help, and the vegetations and soft thrombi or emboli are aspirated [15]. The blood goes through a filter and is then reinfused back to the body with a reinfusion cannula to minimize the blood loss.

The suction filtration and veno-venous bypass system have been primarily used for the removal of large thrombi of the vena cavae, right atrium, and pulmonary arteries as per the published literature [15-16]. Very little data is available regarding its use in RSIE. The idea behind debulking tricuspid valve vegetation is to achieve a lower bacterial load, which will lead to more effective antibiotic efficacy and a lower risk of postoperative recurrence [14]. It also reduces the risk of septic lung embolization and can be used to bridge the surgery to stabilize the patient [13,17]. The data available is limited in terms of demonstrating the use of suction filtration and veno-venous bypass system in RSIE. Hence, more evidence is required to determine the effectiveness of and complications associated with suction filtration and veno-venous bypass system compared to surgery.

## Conclusions

The management of tricuspid valve IE in patients with IVDU remains a challenge. The vast majority of these patients are at a high risk of reinfection. The use of suction filtration and veno-venous bypass system is an alternative therapeutic option if the patient is not a suitable candidate for surgery (such as those who are critically ill with a high risk of perioperative mortality or relapse in the setting of active IVDU). Although the use of suction filtration and veno-venous bypass system is considered a promising noninvasive alternative for the treatment of RSIE, more evidence is required regarding its effectiveness compared to surgery.
